# Une tumeur endobronchique déroutante

**DOI:** 10.11604/pamj.2020.37.201.22896

**Published:** 2020-10-29

**Authors:** Houda Snène, Khalil Zayen, Nozha Ben Salah, Hana Blibech, Hazem Zribi, Ines Marzouk, Mouna Mlika, Leila Ben Farhat, Nadia Mehiri, Béchir Louzir

**Affiliations:** 1 Université de Tunis El Manar, Faculté de Médecine de Tunis, Centre Hospitalier Universitaire Mongi Slim La Marsa, Service de Pneumologie Allergologie, Tunis, Tunisie,; 2 Université de Tunis El Manar, Faculté de Médecine de Tunis, Centre Hospitalier Universitaire Abderrahmen Mami, Service de Chirurgie Thoracique, Ariana, Tunisie,; 3 Université de Tunis El Manar, Faculté de Médecine de Tunis, Centre Hospitalier Universitaire Mongi Slim La Marsa, Service de Radiologie, Tunis, Tunisie,; 4 Université de Tunis El Manar, Faculté de Médecine de Tunis, Centre Hospitalier Universitaire Abderrahmen Mami, Service d´Anatomie Pathologie, Ariana, Tunisie

**Keywords:** Fibroscopie bronchique, tomodensitométrie thoracique, tumeur endobronchique, Bronchial fibroscopy, thoracic CT scan, endobronchial tumor

## Abstract

Le cancer broncho-pulmonaire représente la première cause de décès par cancer chez l´homme et le deuxième chez la femme. Certaines présentations endoscopiques ou radiologiques peuvent orienter le diagnostic histologique et ainsi faciliter la prise en charge thérapeutique. Nous rapportons l´observation d´un homme de 54 ans, tabagique, coronarien récemment stenté, consultant pour hémoptysie et aggravation de sa dyspnée évoluant depuis un mois. Sa radiographie du thorax avait objectivé une hyperclarté de l´hémichamp pulmonaire gauche avec des signes de rétraction. A la fibroscopie bronchique, il existait une formation bourgeonnante framboisée, saignant spontanément, accouchée par la bronche souche gauche évoquant une tumeur carcinoïde. Le scanner thoracique avait objectivé un processus tissulaire endoluminal, au niveau de la bronche souche gauche situé à quatre cm de la carène, peu rehaussé au produit de contraste et compliqué d´atélectasie. Une chirurgie diagnostique et thérapeutique a permis de redresser le diagnostic en faveur d´un hamartochondrome endobronchique.

## Abstract (en)

Bronchopulmonary cancer is the leading cause of death in men and the second in women. Some endoscopic or radiological features may guide histological diagnosis and thus facilitate therapeutic management. We here report the case of a 54-year old man, with a history of smoking and recent coronary stent implantation, presenting with haemoptysis and worsening of dyspnea which had evolved over the last month. Chest x-ray showed left pulmonary hemifield lucency with signs of retraction. Bronchial fibroscopy objectified raspberry bud formation spontaneously bleeding, originating from the left main bronchus and suggesting carcinoid tumor. Chest computed tomography (CT) scan showed poorly enhanced endoluminal tissue process at the level of the left main bronchus, located four cm from the carina and complicated with atelectasis. Diagnostic and therapeutic surgery helped to adjust to a diagnosis of endobronchial amartocondroma.

## Introduction

Les tumeurs endobronchiques sont souvent malignes avec un taux de bénignité qui avoisine 1,9% de toutes les tumeurs pulmonaires [1]. L´hamartochondrome bronchopulmonaire est l´une des tumeurs pulmonaires bénignes les plus fréquentes. Cependant, sa forme endobronchique est rare, représentant 1,4% des hamartochondromes bronchopulmonaires [2]. Dans les cas typiques non compliqués, son diagnostic est facile par le biais de la biopsie bronchique permettant un traitement conservateur grâce à la résection endoscopique. Toutefois, certaines présentations fibroscopiques et radiologiques peuvent faire évoquer une étiologie maligne d´où le recours à une chirurgie diagnostique et thérapeutique. Elles peuvent mimer en tout point une tumeur carcinoïde bronchique qui est considérée comme un néoplasie malin de bas grade composée de cellules neuroendocrines. Ces tumeurs carcinoïdes représentent 1 à 5% de toutes les tumeurs pulmonaires [3]: environ 90% sont bien différenciées appelées «carcinoïdes typiques» et les 10% restantes sont caractérisées histologiquement par une activité mitotique accrue, un pléomorphisme nucléaire et une désorganisation architecturale et sont désignées «carcinoïdes atypiques». Ces dernières ont tendance à avoir un taux de métastase plus élevé et sont plus grandes au moment du diagnostic.

## Patient et observation

**Figure 1:**
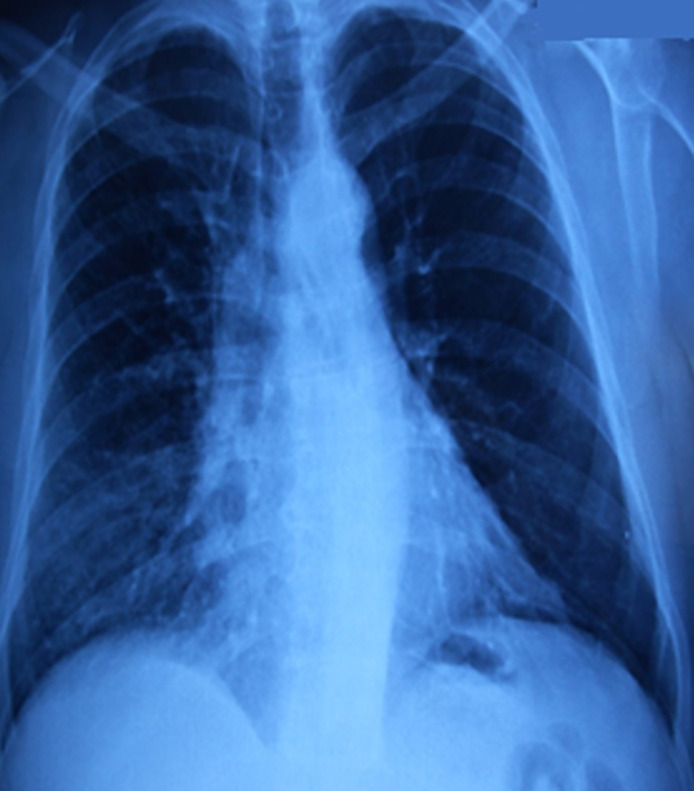
radiographie du thorax de face; élévation de l´hémi-coupole diaphragmatique gauche et hyper-clarté de l´hémichamp pulmonaire gauche

**Figure 2:**
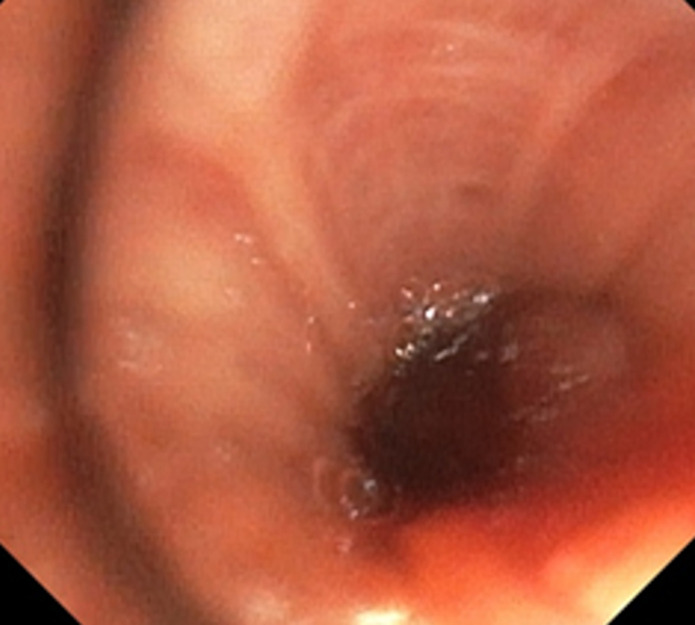
aspect endoscopique; formation bourgeonnante, de couleur rose, saignant spontanément, siège au niveau de la bronche souche gauche

Un homme âgé de 54 ans, tabagique à 40 PA sevré, suivi pour broncho-pneumopathie chronique obstructive, coronarien récemment stenté, sous double antiagrégants plaquettaires, consulte pour hémoptysie et dyspnée d´effort d´aggravation progressive évoluant depuis un mois. A l´examen clinique lors de l´admission, le patient était polypnéique à 23 cycles/min, tachycarde à 97 battements/minutes, présentant des râles sibilants à l´auscultation pulmonaire à gauche avec une saturation périphérique en oxygène à 97%. A la radiographie du thorax, il existait une élévation de l´hémi-coupole diaphragmatique gauche et une inégalité de transparence des deux champs pulmonaires avec un poumon gauche plus clair (Figure 1). La fibroscopie bronchique avait objectivé une formation bourgeonnante, polypoïde, hypervascularisée accouchée dans la lumière de la bronche souche gauche qui est réduite à une fente infranchissable par le fibroscope, à cinq centimètres de la carène, saignant au contact du fibroscope (Figure 2). Cette lésion n´a pas été biopsiée vue le risque hémorragique particulièrement sous double antiagrégants plaquettaires. La cytologie du liquide bronchique était riche en éléments métaplasiques et parakératosiques dont certains contenant des noyaux hyperchromatiques.

**Figure 3:**
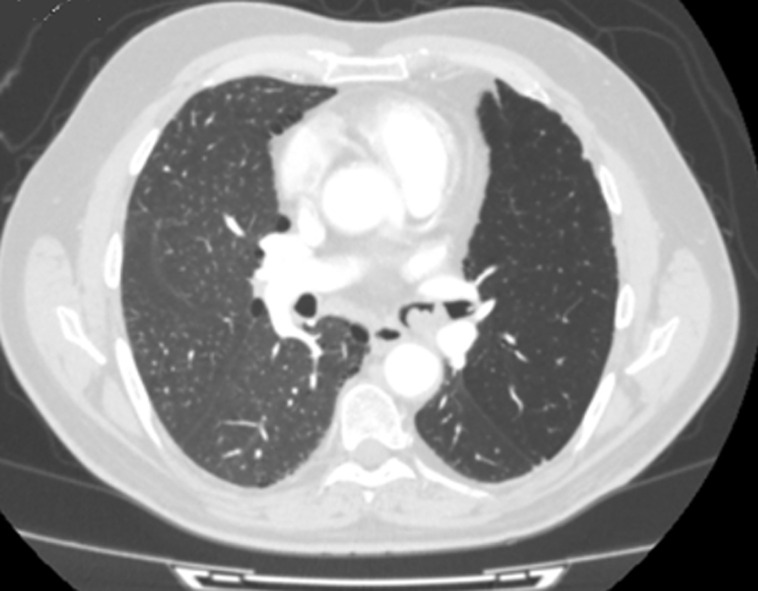
scanner thoracique en fenêtre parenchymateuse avec injection de produit de contraste (PDC); processus endobronchique polylobé au dépend de la bronche souche gauche peu rehaussé après injection de PDC; piégeage aérique de tout le lobe supérieur gauche

**Figure 4:**
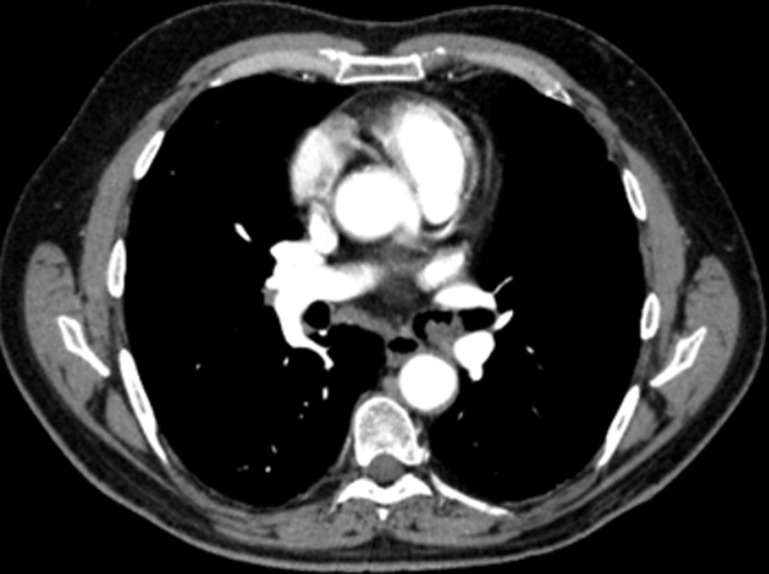
scanner thoracique en fenêtre médiastinale avec injection de produit de contraste (PDC); processus endobronchique polylobé au dépend de la bronche souche gauche peu rehaussé après injection de PDC

La tomodensitométrie (TDM) thoracique avait objectivée un processus tissulaire endoluminal, de contours polylobés situé à 4cm de la carène développée au dépend de la bronche souche gauche mesurant 15x27 mm, peu rehaussé au produit de contraste, venant au contact de l´artère lobaire inférieur gauche et la veine pulmonaire supérieur gauche. Ce processus était compliqué par une obstruction partielle responsable de piégeage aérique de tout le lobe supérieur gauche (Figure 3, Figure 4). Il existait, par ailleurs, des adénomégalies peu suspectes au niveau des chaines médiastinales antérieure, sous carinaire, hilaires et interbronchiques bilatérales. Une chirurgie diagnostique et thérapeutique a été indiquée particulièrement devant l´absence d´endoscopie interventionnelle dans nos différentes structures hospitalo-universitaires. Le patient a été opéré trois mois après la date de son angioplastie pour pouvoir arrêter sa double antiagrégation plaquettaires et l´examen anatomopathologique de la pièce de résection chirurgicale a conclu à un hamartochondrome endobronchique avec des ganglions médiastinaux inflammatoires. Les suites opératoires étaient simples et le patient va bien après un recul de deux ans.

## Discussion

L´hamartochondrome bronchopulmonaire est une tumeur bénigne dérivée du tissu mésenchymateux péribronchique. Elle est constituée de plusieurs composantes tissulaires à proportions variables: du cartilage, de la graisse, du tissu de jonction, de l´épithélium respiratoire et du muscle lisse [4]. Il s´agit d´une tumeur ayant une prédominance masculine avec un âge moyen de survenu entre 60 et 70 ans [2, 5]. Au stade de début de son développement, elle est asymptomatique pour ensuite se manifester, selon sa taille et sa localisation, par des signes d´irritation à type d´hémoptysie ou de toux persistante ou des signes d´obstruction tels qu´une dyspnée, des sibilants ou une atélectasie comme a été le cas de notre patient.

A l´endoscopie, cette tumeur siège habituellement à l´origine des bronches de gros calibre, apparaissant comme une lésion centrale, bien circonscrite, polypoïde, sessile ou pédiculée, à surface lisse jaunâtre sans signes d´infiltrations de la sous-muqueuse. Cette présentation est différente de celle des tumeurs carcinoïdes qui apparaissent comme une lésion rose ou rouge du fait de sa vascularisation [4, 6]. Cependant, la distinction entre un hamartochondrome endobronchique et une tumeur carcinoïde ou toute autre tumeur bénigne reste difficile à l´endoscopie. En effet, comme ça été le cas de notre patient, le bourgeon retrouvé à la fibroscopie était rouge saignant spontanément évoquant en premier lieu une tumeur carcinoïde.

L´aspect scannographique est variable en fonction de la nature du tissu prédominant dans l´hamartochondrome. Certains aspects comme de la graisse intra-tumorale, la faible prise de contraste ou des calcifications en «pop-corn» ou diffuses à la totalité de la masse sont en faveur de la bénignité de la lésion. En effet, l´hamartochondrome endobronchique peut contenir des collections graisseuses (qui apparaissent hypodense à la TDM) isolées ou associées à des foyers de calcifications. Ces aspects sont spécifiques mais rares. La TDM peut aussi mettre en évidence les complications secondaires à l´obstruction bronchique tels que les atélectasies et les foyers de surinfections ou de dilatation de bronches ou de destruction parenchymateuse [7]. Des adénopathies médiastinales réactionnelles peuvent apparaître faisant redouter la malignité. Le PET scan aide généralement au diagnostic en montrant peu ou pas de fixation, mais parfois il peut mettre en évidence un hypermétabolisme. Dans notre cas, une exploration par PET scan aurait peut-être permis d´évoquer le diagnostic mais cette technique reste très difficile d´accès dans notre pratique quotidienne.

Le diagnostic de certitude est histologique, mais il est parfois impossible même avec des biopsies endobronchiques. Devant la suspicion de malignité, une résection chirurgicale devient alors nécessaire allant d´une simple lobectomie à une pneumonectomie [8]. C´est le cas particulièrement des tumeurs carcinoïdes qui nécessitent une étude anatomopathologique sur pièce de résection pour faire la part entre carcinoïde typique et carcinoïde atypique qui ont des pronostics totalement différents [9]. Dans le cas de notre patient, les biopsies bronchiques étaient impossibles à réaliser sous traitement antiagrégant plaquettaire qui ne pouvait pas être arrêté dans les suites d´une angioplastie récente.

Du fait de sa nature bénigne, le traitement de l´hamartochondrome endobronchique est conservateur basé sur la résection endoscopique [1, 10]. Cependant, si la taille de la tumeur est importante ou s´il existe une destruction du parenchyme pulmonaire, la résection chirurgicale est indiquée [10]. Dans notre cas, la progression de la tumeur semble prédominer en intraparenchymateux plus qu´en endobronchique ce qui explique le retard de l´apparition de la symptomatologie; et l´absence d´endoscopie interventionnelle dans nos établissements publics était l´argument décisif pour le choix d´une chirurgie diagnostique et thérapeutique. Dans le cadre du suivi de ces patients traités pour hamartochondrome endobronchique, il existe peu ou pas de risque de transformation maligne et un faible risque de récidive [1, 2].

## Conclusion

L´hamartochondrome endobronchique est une tumeur bénigne qui peut être grave du fait de ses complications (obstruction, hémorragie). Sa prise en charge doit être précoce évitant ces complications et la destruction du parenchyme pulmonaire. Son diagnostic est parfois retardé à cause de sa symptomatologie non spécifique d´où l´intérêt de la fibroscopie bronchique en cas de doute d´un processus endobronchique. Si l´aspect endoscopique et scannographique sont typiques, une biopsie endoscopique est suffisante pour la confirmation diagnostic et le traitement sera alors endoscopique. En cas de présentations atypiques ou de destruction parenchymateuse pulmonaire, le traitement sera chirurgical.
